# Effect of Pt Nanoparticles on the Photocatalytic Activity of ZnO Nanofibers

**DOI:** 10.1186/s11671-015-1126-6

**Published:** 2015-12-16

**Authors:** Alessandro Di Mauro, Massimo Zimbone, Mario Scuderi, Giuseppe Nicotra, Maria Elena Fragalà, Giuliana Impellizzeri

**Affiliations:** CNR-IMM MATIS, Via S. Sofia 64, I-95123 Catania, Italy; CNR-IMM, Zona industriale strada VIII n.5, I-95121 Catania, Italy; Dipartimento di Scienze Chimiche and INSTM UdR Catania, Università di Catania, Viale Andrea Doria 6, I-95100 Catania, Italy

**Keywords:** ZnO nanofibers, Pt nanoparticles, Electrospinning, Photocatalysis

## Abstract

For this study, we originally realized ZnO nanofibers (∼50 nm in mean radius) mixed with Pt nanoparticles (∼30 nm in mean radius), prepared by pulsed laser ablation in liquid, and investigated their photocatalytic performance. The material was synthesized by the simple electrospinning method coupled with subsequent thermal treatments. Methylene blue was employed as a representative dye pollutant to evaluate the photocatalytic activity of the nanofibers. It was found that the Pt-ZnO fibers exhibit a photodegradation reaction rate that is ∼40 % higher than the one obtained for reference ZnO fibers. These encouraging results demonstrate that Pt-ZnO nanofibers can be fruitfully applied for environmental applications.

## Background

Today one of the most pervasive problems afflicting people is inadequate access to clean water and sanitation. The United Nations predicted that by 2030 47 % of the world population will live in areas with high water stress. In recent years, semiconductor photocatalytic process has shown a great potential as a low-cost, environmentally friendly, and sustainable water treatment technology. The ability of semiconductor photocatalysts has been widely demonstrated to remove persistent organic compounds and microorganisms in water [1, 2].

In this field, zinc oxide represents a promising photocatalyst for its high catalytic activity, low cost, and environmental friendliness [3, 4]. When irradiated with ultraviolet (UV) light, the ZnO can mineralize the water pollutants into non-toxic compounds, such as CO_2_, and water [5]. The photocatalytic activity of nanomaterials depends on their size and shape. In particular, smaller nanomaterials are more active compared to their larger counterparts, due to the higher surface area exposed to the polluted water [6]. Thus, a great deal of effort has been devoted to synthesize ZnO nanostructures, including nanowires [7], nanobelts [8], nanorods [9], and nanohelices [10]. Among different methods commonly used to synthesize ZnO nanomaterials [11–13], electrospinning represents a simple and versatile method for producing fibular mesostructures [14, 15].

In a typical electrospinning process, a polymer solution or a melt is injected from a small nozzle under an electric field as strong as several kV/cm. The buildup of electrostatic charges on the surface of a liquid droplet induces the formation of a jet, which is subsequently stretched to form a continuous ultrathin fiber. Earlier reports on electrospun ZnO nanofibers were related to a ZnO precursor in a solution of polyvinyl alcohol (PVA) [16, 17], polyvinylpyrrolidone (PVP) [18], or polyvinylacetate (PVAc) [19].

One of the main drawbacks of photocatalytic semiconductors, limiting their application in photocatalytic process at a large scale, is the high recombination of photogenerated electron-hole pairs. Various strategies have been developed to prolong the lifetime of photo-excited charge carriers, such as the use of noble metal nanoparticles. Thanks to the formation of a Schottky junction at the metal/semiconductor interface, electrons can be efficiently trapped by noble metals, enhancing the electron-hole separation [20, 21]. Recent success in using electrospun nanofibers as supports for metal nanoparticles has been demonstrated for a number of reactions [22, 23]. In detail, the nanofibers of ZnO were usually decorated with metal nanoparticles after the electrospinning process. It is well known that in the case of metal nanoparticles deposited on top of the semiconductor, the photocatalytic efficiency of the system is driven by a compromise between the metal action in capturing the electrons and the coverage of the semiconductor surface resulting from the presence of metal particles that negatively affect the photocatalytic performance of the material [21, 24, 25].

In this paper, we investigate ZnO nanofibers mixed with Pt nanoparticles. The material was originally synthesized by adding Pt nanoparticles, produced by laser ablation, within the polymeric solution before the electrospinning process. The fabrication of zinc oxide nanofibers mixed with platinum nanoparticles (Pt-ZnO) was consequently obtained by a single-step process. The photocatalytic performance of the investigated materials in the photodegradation of methylene blue (MB) was tested in detail.

## Methods

The synthesis of Pt nanoparticles was performed by pulsed laser ablation in liquid method. Nanoparticles were obtained by using the first harmonic (1064-nm wavelength and 10-ns pulse duration) of a Nd:YAG (Giant G790-30) Q-switched laser. The laser beam was focused through a lens (focal length 20 cm) on a pure platinum target (Sigma Aldrich, purity 99.9 %) placed on the bottom of a Teflon vessel filled with 2 ml of de-ionized water (MilliQ, resistivity 18 MΩ⋅cm). The pulse energy was 337 mJ/pulse, and the fluence was estimated to be approximately 5 J/cm^2^.

The as-prepared nanoparticles were characterized by dynamic light scattering (DLS) technique. DLS measurements were performed by means of a homemade apparatus as described elsewhere [26], and the analysis of the DLS spectra was performed by using the II cumulant method. The hydrodynamic diameter and polydispersity index were estimated to be 70 ± 2 nm and 0.18, respectively.

The fabrication of nanofibers was achieved by electrospinning: a solution containing 1 g of PVP (medium molecular weight = 1,300,000 Da), and 0.36 g of zinc acetate (Zn(CH_3_COO)_2_ · H_2_O) in 2 ml of dimethylformamide (DMF) (all reagents were purchased from Sigma Aldrich), was added to 1 ml of Pt nanoparticles solution, in order to obtain a solution with 1 % of Pt nanoparticles. The resulting solution was electrospun using a commercial electrospinning apparatus (EC-DIG Electrospinning, IME Technologies). The composite was ejected from the needle (21 gauge) of a syringe applying an electrical field as high as several kV/cm. Nanofibers were collected (stationary mode) on the surface of silicon substrates clamped on top of a conductive circular collector. Some ZnO nanofibers without Pt were electrospun as reference. Electrospinning parameters are reported in Table 1.

**Table 1:** Electrospinning conditions for the deposition of ZnO and Pt-ZnO nanofibers

	Collector	Distance (cm)	Flow rate (μl/min)	Negative voltage (kV)	Positive voltage (kV)	Time (min)
ZnO	Silicon	16.0	5.0	−4	18	3
Pt-ZnO	Silicon	17.0	5.3	−4	20	3

With the purpose of synthesizing nanofibers with similar radii, it was necessary to opportunely modulate the distance between the needle and the Si substrates, and the positive applied voltage, because the addition of water (with dissolved Pt nanoparticles) in the Zn precursor solution causes a change in the viscosity and superficial tension of the solution. Different tests were performed, modifying the mean Pt radius, the water/DMF ratio, and the electrospinning parameters, to optimize the morphological properties of the synthesized nanofibers. In order to remove the polymer after the electrospinning process, the composites were annealed at different temperatures, from 350 to 500 °C, for 1 h, in a conventional furnace under a controlled oxygen atmosphere (O_2_ flow: 2.5 l/min).

The morphological characterization was performed by scanning electron microscopy with a field emission (FE-SEM) Zeiss Supra 25 and by transmission electron microscopy (TEM) through a Cs-probe-corrected JEOL ARM200CF at a primary beam energy of 200 keV, operated in scanning mode (STEM) and equipped with a 100-mm^2^ SDD energy dispersive X-ray (EDX) detector.

The crystallinity was analyzed by X-ray diffraction (XRD) analyses with a Bruker D-500 diffractometer (detector scan mode) at 0.5° angle of incidence and 2*θ* from 20 to 60°. The XRD spectra were analyzed by the Bruker software suite, including the ICSD structure database.

The photocatalytic activity of the investigated materials was tested by the degradation of MB, complying with the ISO protocol [27]. Before any measurement, the samples were irradiated by an UV lamp for 60 min in order to remove the hydrocarbons from the sample surface [28]. The samples, 1 cm × 1 cm in size, were immersed in a 2 ml solution containing MB and de-ionized water, with a starting concentration of MB of 1.5 × 10^−5^ M. The measurements were performed at a fixed pH value (7.5), using NaOH. The mixture was irradiated by an UV lamp (350 to 400 nm wavelength range) with an irradiance of 1.1 mW/cm^2^ (which simulates the UV irradiance of the sun on the Earth). The irradiated solution was measured at regular time intervals with an UV-VIS spectrophotometer (Lambda 35, Perkin-Elmer) in a wavelength range of 500–800 nm. The degradation of MB was evaluated by the absorbance peak at 664 nm in the Lambert-Beer regime [29]. The decomposition of the MB dye in the absence of any photocatalyst materials was checked as a reference. Control experiments in the dark for 60 min were conducted to clarify the contribution of the adsorption of the MB at the sample surface.

The photocatalytic activity of the nanofibers (with and without Pt) was compared with that of a flat film of ZnO. The film was deposited on a Si substrate by atomic layer deposition (ALD), with a Picosun R-200 advanced system. During the deposition, the temperature was kept at 300 °C. The two precursors were diethyl zinc and de-ionized water, while N_2_ was used as a carrier and purge gas. The film thickness (∼30 nm) was evaluated by the M-2000 spectroscopic ellipsometer by Woollam.

## Results and Discussion

**Fig. 1:**
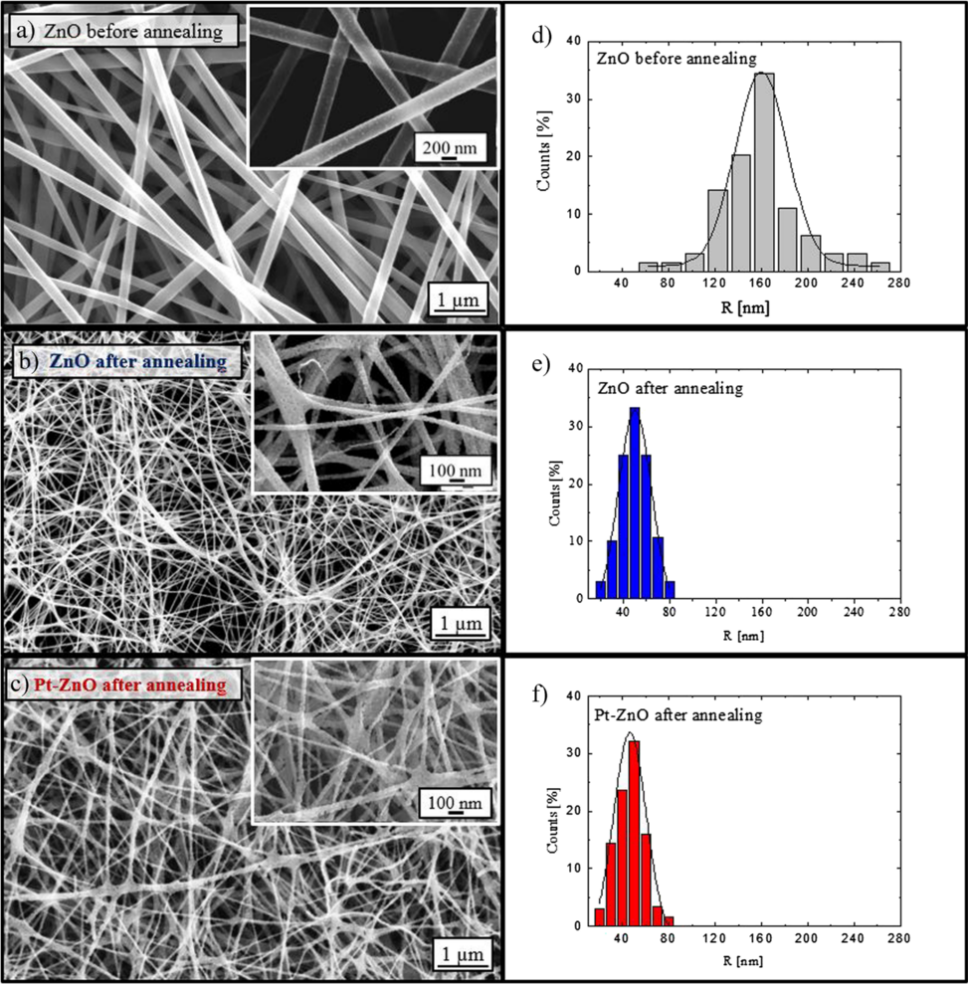
SEM images of ZnO and Pt-ZnO nanofibers. **a–c** Plan-view SEM images (*inset*: higher magnification images) and **d–f** radius distributions for each sample

Figure 1 shows the plan-view SEM images of high density ZnO nanofibers before the calcination (panel a) and after the calcination at 500 °C without (panel b) or with Pt nanoparticles (panel c). Before the SEM analyses, only the sample analyzed before the calcination was metalized in order to reduce the charge effect. The synthesized nanofibers have a random orientation, as expected due to the instability of the electrospinning jet [15]. The size distribution of the nanofibers has been calculated by counting the number of nanofibers with the same radius, by using Digital Micrograph 3.6.1 (Gatan Inc.), by taking into account all visible nanofiber boundaries and subtracting half of those nanofibers that intersect an edge. The radii distributions reported in Fig. 1d–f for the ZnO nanofibers before calcination and ZnO and Pt-ZnO nanofibers after calcination, respectively, indicate a Gaussian distribution around a radius mean value. The mean radius of the nanofibers has been calculated assuming cylindrical-shaped nanofibers. Before the calcination process, the mean fiber radius was 156 ± 37 nm. As a consequence of the calcination process, the nanofibers significantly shrunk because of the removal of the PVP component. The morphology of nanofibers was not influenced by the aqueous solution of platinum nanoparticles added at the polymeric solution; indeed, the resulting mean fiber radius was 48 ± 10 nm, regardless of the presence of Pt nanoparticles in the solution.

**Fig. 2:**
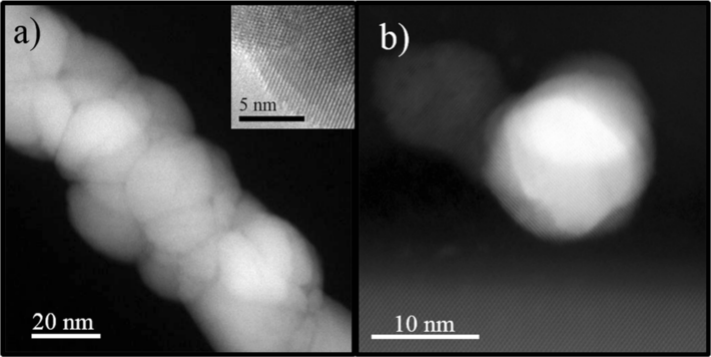
HR-TEM and STEM Z-contrast images of ZnO nanofibers and Pt nanoparticles. Z-contrast STEM images of ZnO nanofibers (**a**), bright field HR-TEM on ZnO nanofibers (*inset*), and atomic-resolution STEM cross section of the sample showing a Pt nanoparticle on the Si substrate (**b**)

**Fig. 3:**
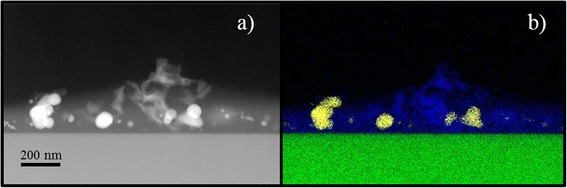
STEM and 2D elemental chemical EDX map of Pt-ZnO nanofibers. Z-contrast STEM image of a cross section of the sample showing Pt nanoparticles and ZnO nanofibers (brighter contrast regions came from Pt particles) (**a**) and the corresponding 2D elemental chemical EDX map showing Zn (*blue*), Pt (*yellow*), and Si (*green*) (**b**)

A more detailed morphological characterization was obtained by Z-contrast STEM analyses. The ZnO nanofibers appear as an agglomeration of ZnO grains (Fig. 2a). The inset in Fig. 2a shows a high-resolution TEM (HR-TEM) image of a ZnO nanofiber in which we can see the crystal planes of the ZnO domain. An atomic resolution of Pt nanoparticles, reported in Fig. 2b, indicated a crystalline structure of the nanoparticles. Thus, high-resolution and atomic-resolution images revealed the crystalline nature of the Zn nanofibers and Pt nanoparticles, too. The Pt nanoparticles clearly appear to be separated and mainly located in proximity of the substrate and among the ZnO nanofibers. The corresponding experimental 2D chemical EDX map of Fig. 3a is reported in false colors in Fig. 3b. Here the Zn is indicated in blue and the Pt in yellow, while the Si from the substrate is green. Therefore, we synthesized with a single-step process a Pt-ZnO nanosystem, with ZnO nanofibers and Pt nanoparticles in between. This happens due to the interaction of zinc acetate and Pt with PVP. During the calcination process, the zinc interacts with oxygen so as to form ZnO, while the Pt particles lose their coordination with the polymeric matrix locating among the nanofibers. Consequently, with this simple process, we distributed Pt nanoparticles among the nanofibers, potentially improving the photocatalytic activity of the mere ZnO nanofibers.

**Fig. 4:**
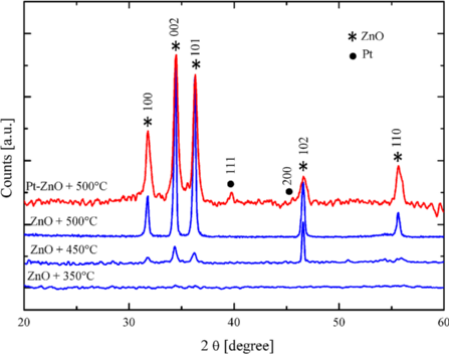
XRD patterns of ZnO nanofibers. ZnO nanofibers calcinated at different temperatures (*blue lines*) and Pt-ZnO nanofibers calcinated at 500 °C (*red line*)

The fibers were deeply characterized by XRD analyses. Figure 4 shows the patterns of the ZnO nanofibers calcinated at different temperatures (from 350 to 500 °C) and the pattern of the Pt-ZnO nanofibers calcinated at the optimized temperature of 500 °C. The spectra clearly show that the ZnO fibers calcinated at 350 °C are mainly amorphous. Well-defined features appear for a calcination temperature of 450 °C, due to the crystallization of the zinc oxide. In particular, five main peaks appear, associated to the following crystallographic planes: (100), (002), (101), (102), and (110), marked with “*” in Fig. 4, which characterize the hexagonal wurtzite structure of ZnO (*a* = 3.249 Å, *c* = 5.206 Å) [15]. The diffraction data are in good agreement with the Joint Committee on Powder Diffraction Standards (JCPDS) card of ZnO [JCPDS 36-1451]. The diffraction peaks confirm that in our system, the calcination above 450 °C was sufficient to decompose ZnO/PVP so as to obtain polycrystalline ZnO nanofibers. In the diffraction pattern of the Pt-ZnO, two additional peaks, corresponding to the crystallographic planes (111) and (200) of Pt, are detected, marked with “•” in Fig. 4. Thus, XRD analyses confirmed the crystalline structure of both the ZnO nanofibers and Pt nanoparticles, previously evidenced by high-magnification STEM images.

**Fig. 5:**
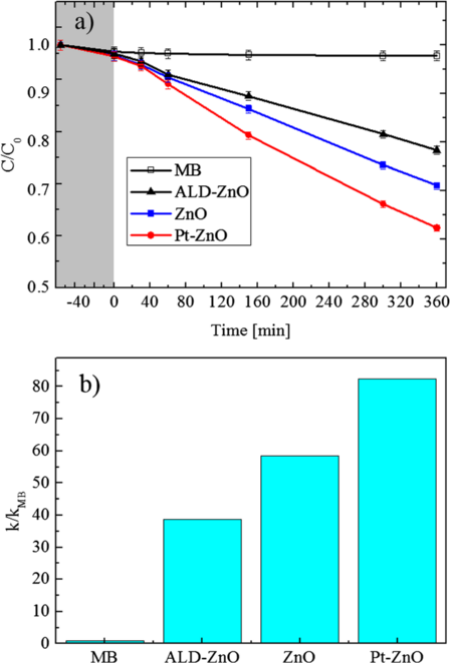
Photodegradation activity of ZnO and Pt-ZnO nanofibers. **a** MB degradation under UV irradiation for four samples: MB (*open squares*), MB with the ZnO thin film (*closed triangles*), MB with the ZnO nanofibers (*closed squares*), and Pt-ZnO nanofibers (*closed circles*). **b** Photodegradation reaction rate, normalized to the value obtained for the MB in the absence of the catalyst, for the different investigated samples: ALD-ZnO flat film, ZnO, and Pt-ZnO nanofibers

The photocatalytic activity of the nanofibers was evaluated by the degradation of methylene blue dye under UV irradiation. The photodegradation of MB can be considered as a pseudo first-order reaction, and its kinetics can be expressed as follows:

1 $$ C={C}_0\;{e}^{-kt} $$

where *k* is the degradation reaction rate, and *C*_0_ and *C* are the starting concentrations of MB and the concentration at the reaction time *t*, respectively [1]. The absorbance peak at 664 nm is a direct measurement of the MB concentration (through the Lambert-Beer law), and thus, its decrease with the UV irradiation time is a direct measurement of the photocatalytic decomposition of the MB molecules. The photocatalytic response of the ZnO nanofibers with or without the Pt nanoparticles was compared to the response of the MB solution without any catalyst materials and with a thin flat film of ZnO deposited by ALD. Figure 5a shows the residual concentration, *C*/*C*_0_, of the MB versus the irradiation time. We tested four samples: MB in the absence of any catalyst materials (open squares), MB with a ZnO flat film (closed triangles), MB with a ZnO nanofiber sample (closed squares), and MB with a Pt-ZnO nanofiber sample (closed circles). The first test was performed under dark conditions for 1 h (reported in gray color in Fig. 5a), in order to evaluate the degree of adsorption of the MB on the beaker walls and on the surface of the three types of samples. All the samples did not show any adsorption within the experimental errors (∼1 %). A significant decrease in the MB concentration is clearly observed when the photocatalytic materials are added to the solution. After an UV irradiation for 6 h, the ZnO flat film decomposed about 20 % of the MB. A more important MB decomposition of ∼30 % was observed thanks to the ZnO nanofibers, clearly due to an increase of the exposed surface area with respect to the flat film. The best result was obtained with the Pt-ZnO nanofibers, with a MB decomposition of ∼40 %. Thus, the photocatalytic test showed a significant improvement of the photocatalytic activity due to the Pt presence with a small concentration of 1 % in the starting solution. This improved photo-activity can be attributed to the more efficient charge carrier separation in the Pt-ZnO composite than in the pure ZnO, due to the presence of metal nanoparticles that act as an electron scavenger. As observed by SEM images (not shown), the discoloration test did not change significantly the morphological structures of the nanofibers.

Figure 5b reports the degradation rate of the MB, normalized to the value obtained for the MB decomposition in the absence of any catalyst materials, for all the samples, under UV light irradiation. On the abscissa axis, MB indicates the MB decomposition in the absence of a catalyst that is always 1 due to the normalization done (i.e., *k*/*k*_MB_); ALD-ZnO refers to the MB decomposition in the presence of a thin flat film (∼30 nm in thickness) deposited by ALD; ZnO and Pt-ZnO indicate the MB decomposition in the presence of unloaded and Pt-mixed ZnO nanofibers, respectively. In detail, the photodegradation reaction rate (*k*) resulted to be 6.1 × 10^−4^ min^−1^ for the ZnO flat film, 9.2 × 10^−4^ min^−1^ for pure ZnO nanofibers, and 1.3 × 10^−3^ min^−1^ for Pt-ZnO nanofibers. The samples with 1 % Pt show a significantly higher photocatalytic activity than the samples without Pt, with a photodegradation reaction rate ∼40 % higher than the one obtained for the reference ZnO nanofibers.

Our results indicate a significant improvement of the photocatalytic performance of the ZnO nanofibers enriched with Pt nanoparticles. Several groups investigated the photocatalytic efficiency of Pt/ZnO systems. Zang et al. [24] fabricated a high-efficiency microreactor with Pt-nanoparticle-coated ZnO nanorod arrays on the inner wall, for the degradation of phenol in water. They used an UV lamp with a significantly higher irradiance, ∼23 times higher than ours, and tested the material towards the degradation of phenols. These different experimental conditions (i.e., different irradiance and different pollutant) make difficult a comparison. Chio et al. [25] studied the correlation between the electronic structures and the photocatalytic activities of nanocrystalline Au, Ag, and Pt particles on the surface of ZnO nanorods. The presence of Pt on the ZnO surface generated a greater occupation of the O 2p orbitals and so a greater negative effective charge of the O ions than Au and Ag nanoparticles. This result suggests that Pt nanoparticles have weaker photocatalytic activities than those of Au and Ag nanoparticles. However, it is also worth noting that an excessive coating of the ZnO surface by metal nanoparticles, reducing the surface sites available for the water purification process, negatively affects the photocatalytic performance of the semiconductor. Finally, Zeng et al. [30] reported a study about the fabrication of oxide-based hollow nanoparticles using core/shell nanostructures of active metal/oxide nanoparticles. The material was tested towards the degradation of the methyl orange. The presence of a noble metal (Au or Pt), inside the core shell, causes a 75 % improvement of the photocatalytic activity with respect to pure ZnO nanospheres. This great result, if compared with ours, is influenced by the high amount of the used photocatalyst (10 mg in 20 ml of dye solution) and the high lamp power employed for the experiment (∼16 times higher than ours). Albeit a comparison with the existing literature remains difficult, it is clear that the photocatalytic material presented in this paper is effective and advantageous. In fact, the method of synthesis is simple, versatile, scalable, and commonly applied in textile and filters factories, and we have demonstrated how even a minimum amount of Pt nanoparticles is enough to significantly enhance the photocatalytic activity.

## Conclusions

In conclusion, we synthesized electrospun ZnO nanofibers (∼50 nm in mean radius) eventually mixed with Pt nanoparticles (∼30 nm in mean radius). The addition of Pt nanoparticles was proven effective to improve the nanofibers’ photocatalytic activity of ZnO in the degradation of methylene blue. In particular, the Pt-ZnO nanofibers exhibited a photodegradation reaction rate that is ∼40 % higher than that observed for mere ZnO nanofibers. The fibrous structure, which facilitates the contact of nanofibers with the dye, together with the presence of Pt, which improves the separation of charge carriers, makes Pt-ZnO a promising candidate for photocatalytic applications.

The methodology described in this article offers a simple and versatile route to fabricate Pt-ZnO nanofibers, attractive for a wide range of applications such as water purification, air purification, self-cleaning, gas sensing, and hydrogen production by water splitting.

## Abbreviations

DMF: dimethylformamide
EDX: energy dispersive X-ray
FE-SEM: field emission scanning electron microscopy
JCPDS: Joint Committee on Powder Diffraction Standards
MB: methylene blue
Pt-ZnO: zinc oxide nanofibers mixed with platinum nanoparticles
PVA: polyvinyl alcohol
PVAc: polyvinylacetate
PVP: polyvinylpyrrolidone
STEM: scanning transmission electron microscopy
UV: ultraviolet
XRD: X-ray diffraction
